# XGboost Prediction Model Based on 3.0T Diffusion Kurtosis Imaging Improves the Diagnostic Accuracy of MRI BiRADS 4 Masses

**DOI:** 10.3389/fonc.2022.833680

**Published:** 2022-03-17

**Authors:** Wan Tang, Han Zhou, Tianhong Quan, Xiaoyan Chen, Huanian Zhang, Yan Lin, Renhua Wu

**Affiliations:** ^1^ Radiology Department, Second Affiliated Hospital of Shantou University Medical College, Shantou, China; ^2^ Institute of Health Monitoring, Inspection and Protection, Hubei Provincial Center for Disease Control and Prevention, Wuhan, China; ^3^ Department of Electronic and information Engineering, College of Engineering, Shantou University, Shantou, China; ^4^ Guangdong Provincial Key Laboratory for Breast Cancer Diagnosis and Treatment, Cancer Hospital of Shantou University Medical College, Shantou, China

**Keywords:** breast cancer, BiRADS 4, diffusion kurtosis imaging, imaging marker, XGboost model

## Abstract

**Background:**

The malignant probability of MRI BiRADS 4 breast lesions ranges from 2% to 95%, leading to unnecessary biopsies. The purpose of this study was to construct an optimal XGboost prediction model through a combination of DKI independently or jointly with other MR imaging features and clinical characterization, which was expected to reduce false positive rate of MRI BiRADS 4 masses and improve the diagnosis efficiency of breast cancer.

**Methods:**

120 patients with 158 breast lesions were enrolled. DKI, Diffusion-weighted Imaging (DWI), Proton Magnetic Resonance Spectroscopy (^1^H-MRS) and Dynamic Contrast-Enhanced MRI (DCE-MRI) were performed on a 3.0-T scanner. Wilcoxon signed-rank test and χ2 test were used to compare patient’s clinical characteristics, mean kurtosis (MK), mean diffusivity (MD), apparent diffusion coefficient (ADC), total choline (tCho) peak, extravascular extracellular volume fraction (V_e_), flux rate constant (K_ep_) and volume transfer constant (K^trans^). ROC curve analysis was used to analyze the diagnostic performances of the imaging parameters. Spearman correlation analysis was performed to evaluate the associations of imaging parameters with prognostic factors and breast cancer molecular subtypes. The Least Absolute Shrinkage and Selectionator operator (lasso) and the area under the curve (AUC) of imaging parameters were used to select discriminative features for differentiating the breast benign lesions from malignant ones. Finally, an XGboost prediction model was constructed based on the discriminative features and its diagnostic efficiency was verified in BiRADS 4 masses.

**Results:**

MK derived from DKI performed better for differentiating between malignant and benign lesions than ADC, MD, tCho, K_ep_ and K^trans^ (*p < 0.05*). Also, MK was shown to be more strongly correlated with histological grade, Ki-67 expression and lymph node status. MD, MK, age, shape and menstrual status were selected to be the optimized feature subsets to construct an XGboost model, which exhibited superior diagnostic ability for breast cancer characterization and an improved evaluation of suspicious breast tumors in MRI BiRADS 4.

**Conclusions:**

DKI is promising for breast cancer diagnosis and prognostic factor assessment. An optimized XGboost model that included DKI, age, shape and menstrual status is effective in improving the diagnostic accuracy of BiRADS 4 masses.

## Introduction

Breast cancer (BC) is the most common cancer and a leading cause of female mortality worldwide (1). It presents substantial heterogeneity in histology, clinical presentation and therapy response. Four major BC subtypes can be defined by gene expression profiling: luminal A, luminal B, HER2-enriched, and basal-like (triple-negative BC, TNBC) (2, 3). Among patients with BC, recurrence-free and overall survival are thought to be related to the histological grade, Ki-67 expression, the status of lymph node (LN), estrogen receptor (ER), progesterone receptor (PR), and human epidermal growth factor receptor 2 (HER2) (4).

Magnetic resonance imaging (MRI), a non-invasive modality that provides an excellent soft-tissue contrast with high sensitivity, is well-established for BC characterization, treatment planning, and post-operative prognostication (5). Dynamic contrast-enhanced MRI (DCE-MRI), which enables detailed morphologic and haemodynamics evaluations through pharmacokinetic modeling techniques, has been widely used for BC diagnosis and monitoring tumor’s response to chemotherapy (6–10). However, the diagnostic specificity of DCE-MRI for BC varies greatly due to background parenchymal enhancement and overlapping of the time-intensity curves between benign and malignant lesions, which leads to unnecessary biopsies (8). Furthermore, DCE-MRI may not be appropriate for patients who are allergic to contrast agents or have liver or kidney dysfunction. *In vivo* proton MR spectroscopy (^1^H-MRS) provides molecular and biochemical information on tumor classification based on the observation of total choline (tCho) levels (11). However, tCho provides limited sensitivity for differentiation between breast lesion types (12). Apparent diffusion coefficient (ADC) derived from diffusion-weighted imaging (DWI) assumes an ideal Gaussian distribution of water displacement without any restriction (13, 14), it’s another non-contrast MRI modality to assess complex tissue microstructural features (15, 16). However, water diffusion in living tissues is generally restricted due to the complex microstructural environment, including the presence of cell membranes and other organelles, and thus tends to deviate from a Gaussian distribution (17). Diffusion kurtosis imaging (DKI) follows a non-Gaussian distribution and is considered useful for characterizing heterogeneous tumors. This modality was introduced by Jensen et al., and included parameters of mean kurtosis (MK) and mean diffusivity (MD) (18). In this context, greater MK or lower MD values suggest more restrictions to normal water diffusion and greater tissue complexity (19). Previous DKI applications revealed its greater sensitivity over ADC for characterizing hepatocellular carcinoma, glioma, BC, and prostate cancer (20–23). Sun et al. observed using 1.5-T imaging on BC that greater MK was significantly associated with higher histological grade and elevated Ki-67 expression (24). Similarly, our preliminary work on 3.0-T MRI revealed the usefulness of MK for breast lesions (BLs) characterization (25).

Breast MRI findings using the Breast Imaging Reporting and Data System (BiRADS) lexicon descriptors provide a standardized language to define the final assessment categories for predicting the likelihood of malignancy, allowing radiologists to communicate important findings in a consistent and repeatable manner (26, 27). However, the malignant probability of MRI BiRADS 4 ranges from 2% to 95% (28), leading to unnecessary biopsies and even causing huge anxiety for patients.

XGboost is a more powerful version of the Gradient Boosting Decision Tree (GBDT) algorithm (29), in which a second-order Taylor expansion is performed on the square loss function to achieve better accuracy.The objective function is defined as follows:


$${\text{Obj}}^{\left(\text{t}\right)}≅\sum_{i=1}^{n}\left[g_{i}f_{i}(x_{i})+\frac{1}{2}h_{i}f_{t}^{2}(x_{i})]+\text{Ω}(f_{t}\right)$$

$$\text{where Ω}(f_{t})=\gamma T+\frac{1}{2}\lambda \sum_{j=1}^{T}w_{j}^{2}$$

Here g*_i_* and *h_i_* are first and second order gradient statistics on the loss function. *n* represents the numbers of samples. *f_t_* (*x_i_*) represents the regression tree functions at the t-th iteration. *T* represents the number of leaves in the tree. $w_{j}^{2}$ represents L2 norm of leaf scores. Ω(*f_t_*) the regularization term based on complexity of the model (i.e. number and weight of leaf nodes), which effectively prevents overfitting. In addition, XGboost adopts shrinking and column subsampling techniques to improve the generalization and learning speed of the algorithm. These advantages make XGboost a widely accepted model in many machine learning and data mining applications (30, 31).

Given that MK derived from DKI is a promising imaging marker for predicting the aggressiveness of tumors according to previous preliminary studies (24, 25), and XGboost is a scalable machine learning system for tree boosting. Thus, the purpose of this study involved in larger sample size was to construct an optimal XGboost prediction model through a combination of DKI independently or jointly with other MR imaging features and clinical characterization, which was expected to reduce the false positive rate of MRI BiRADS 4 and improve diagnosis efficiency of BC. So far, no study has reported that the XGboost model based on DKI improves the diagnostic specificity of MRI BiRADS 4. This information could help radiologists provide referring clinicians with a promising prediction model to increase the diagnostic accuracy of MRI BiRADS 4, thereby preventing unnecessary biopsies and optimizing personalized diagnosis and treatment.

## Material and Methods

### Patients

**Figure 1:**
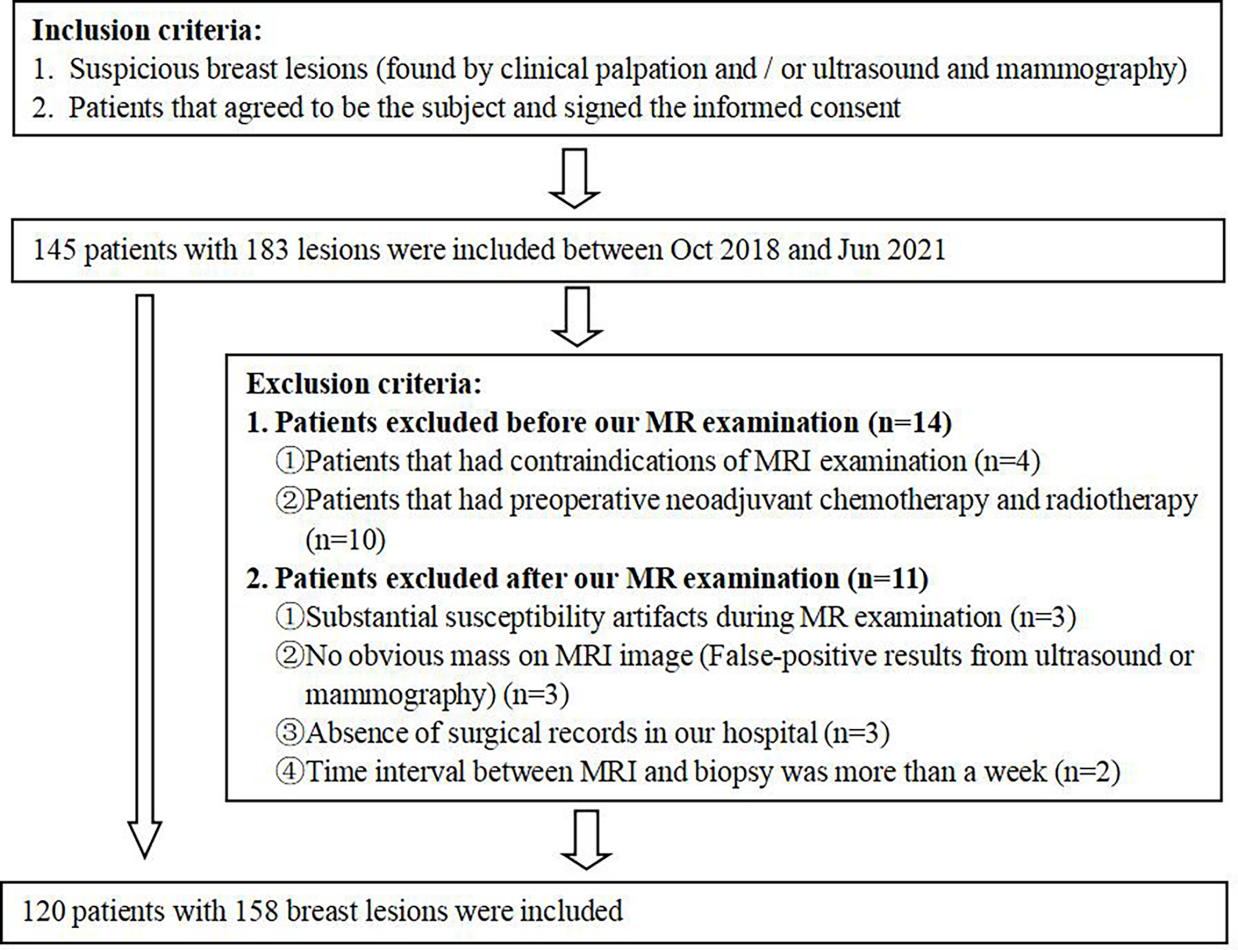
Flowchart of the study population.

This study protocol was approved by our Institutional Review Board, and written informed consent was obtained from all patients. 120 patients (median age: 44 years, range: 17–71 years) with 158 BLs were recruited from the Department of General Surgery of our hospital between October 2018 and June 2021. Patients were excluded based on the flowchart as shown in **Figure 1**. Twenty-two patients had two or more neoplasms, and each lesion was examined separately. 158 BLs were divided into a training group (n=108, malignancy=53, benign=55) and a validation group (n=50, malignancy=25, benign=25). The training group, consisting of 6 BiRADS 2 masses, 56 BiRADS 3 masses, 12 BiRADS 4 masses and 34 BiRADS 5 masses, was used to construct an XGboost diagnostic model. The validation group, included 50 BiRADS 4 masses, was used to verify the diagnostic performance of the XGboost model in BiRADS 4 masses. The BiRADS classification of MRI was evaluated based on the morphology findings, dynamic enhancement pattern and ADC measurement of lesion, according to the American College of Radiology BiRADS 5^th^ version for breast MRI (26, 27).

### MRI Protocol

All MRI examinations were performed using a 3.0-T MR scanner (GE Medical System, Milwaukee, WI, USA) with a dedicated four-channel bilateral breast coil. Premenopausal women were examined in the prone position after the first week of their menstrual cycle. Following a T1-weighted FSE-XL sequence and a T2-weighted FRFSE-XL sequence, the routine DWI and DKI by an echo-planar imaging sequence, ^1^H-MRS as well as DCE imaging were performed. The protocol parameters were shown in **Table 1**. A cubic region of interest (ROI, 1–6 cm^3^) was positioned inside the lesion for ^1^H-MRS acquisition, with 4 saturation bands. An automatic shimming adjustment of the unsuppressed water signal was performed to reach water linewidths of 10–20 Hz.

**Table 1:** Imaging protocol parameters for T_1_WI, T_2_WI, DWI, DKI, ^1^H-MRS and DCE-MRI.

Parameter	T_1_WI	T_2_WI		ADC	DKI	^1^H-MRS	DCE-MRI
Sequence	FSE-XL	FRFSE-XL		DW-EPI	DW-EPI	PRESS	VIbrant
Orientation	Axial	Axial		Oblique Axial	Oblique Axial	Axial	3-dimension
Repetition time (ms)	333	4100		5000	5000	2000	3.9
Echo time (ms)	7.6	76.4		91.0	69.6	155	2.1
Fat suppression	–		Dixon	STIR	STIR	–	SPECIAL
Field of view (cm)	35	35		35	35	35	35
Matrix	320×256	320×224		128×128	128×128	256×192	256×256
Slice thickness (mm)	6	6		6	6	–	5
No. of sections	24	24		48	2024	–	1024
Bandwidth (Hz/pixel)	41.7	83.3		250	250	2.5	83.3
b values (s/mm^2^)	–	–		0, 800	0, 500, 1000, 1500, 2000, 2500	–	–
Number of diffusion directions				3	15		
Total scan time (s)	94	185		200	430	243	326

### MRI Analysis

All raw diffusion imaging data were post-processed using Functool 9 software, which is integrated into the MR imager (GE Medical System, Ruede la Minière, France). This process automatically generates the imaging metrics of DWI and DKI. ADC maps were generated from DWI using b values of 0 and 800 s/mm^2^, considering all 3 diffusion gradient directions. MK and MD maps were derived from DKI using b values of 0, 500, 1,000, 1,500, 2,000, and 2,500 s/mm^2^, considering all 15 diffusion gradient directions. ROIs (mean size: 94 ± 33 mm^2^, range: 60–380 mm^2^) for each lesion were manually drawn on three different solid neoplastic regions while avoiding necrotic tissue, hemorrhagic components, and dominant ducts. Average ADC, MK, and MD values were subsequently calculated. LCModel software (Canada) was used to identify the tCho peak at 3.23 ppm in the breast spectrum inside the lesion. DCE-MRI data were post-processed using GenIQ software integrated into the MR imager. Taking the standard map and modified Tofts model as the mathematical model, the functional maps of volume transfer constant (K^trans^), flux rate constant (K_ep_) and extracellular volume fraction (V_e_) were obtained in at least two-thirds of the breast lesions.

The characteristics of the lesions included size, shape, margin, BiRADS categories and imaging parameters were analyzed by two senior radiologists specialized in breast imaging (5 and 10 years of diagnostic experience) and who were blinded to the histopathological diagnosis. The size of the lesions was measured in MR imager on the last phase in DCE-MRI. Intra-class correlation coefficients (ICCs) were used to assess the consistency of parameters calculated twice by the radiologists, and “good” correlation was defined as an ICC >0.75.

### Pathology and Immunohistochemistry

The histopathological findings were analyzed by two experienced pathologists (5 and 10 years of pathologically diagnosing breast tumors), blinded to the patients’ clinicopathological characteristics. BC histological grades were based on the modified Bloom–Richardson guidelines (grades 1–2: low-grade disease, grade 3: high-grade disease) (32). LN status was determined by the postoperative histopathological examination. Positive results for ER and PR expression were defined as positive staining for ≥1% of nuclei in 10 high-power fields (33). The positive result for HER2 expression was defined as an immunohistochemical result of 3+ or if gene amplification was observed *via* fluorescence *in situ* hybridization (34). The Ki-67 nuclear protein reflects cell proliferation and its expression was scored as the percentage of tumor cell nuclei with positive immunostaining based on a threshold value of 14% (high Ki-67 expression: >14%) (35, 36).

### Statistical Analysis

The Kolmogorov–Smirnov test was initially used to analyze for normal distributions of variables and then the Levene test to examine the homogeneity of variance. Wilcoxon signed-rank test and χ2 test were used to evaluate continuous and categorical variables, respectively. Receiver operating characteristic (ROC) curve analysis was used to evaluate the performances of the imaging parameters for diagnosing BC and predicting histopathological findings, with excellent diagnostic ability defined as an area under the ROC curve (AUC) >0.8. Spearman correlation analysis was used to evaluate the associations between the imaging parameters and the BC prognostic factors and molecular subtypes. Correlations were classified based on the correlation coefficient as excellent (≥0.75), moderate to good (0.50–0.74), fair (0.25–0.49), and small (≤0.24).

All statistical analyses were performed using IBM SPSS (version 25; IBM Corp., Armonk, NY), MedCalc (version 15.6.1; Ostend, Belgium), GraphPad Prism (version 7, GraphPad, USA), and python (version 3.8.8). Differences were considered statistically significant at *p*-values <0.05.

### XGBoost Model Construction and Verification

The XGBoost model was constructed and verified in four stages: I. Significant features were selected based on Wilcoxon signed-rank test and χ2 test. II. The best imaging parameters were selected based on the ROC curve. The optimal combination of imaging parameters and clinical features were selected based on the least absolute shrinkage sum selection operator (LASSO). III. Representative features were used to construct the XGBoost model and to derive feature importance scores, where the optimal feature subset was determined by 3-fold cross-validation performed 50 times. IV. BiRADS 4 lesions in the validation group were used to verify the effectiveness of the model.

## Results

### Clinicopathological Findings

This study evaluated 158 BLs in 120 patients. 78 lesions (49.4%) were confirmed to be malignant, including invasive ductal carcinoma (n=64), invasive lobular carcinoma (n=1), medullary carcinoma (n=3), papilloma carcinoma (n=4), mucinous adenocarcinoma (n=1) and intraductal carcinoma (n=5). The remaining 80 lesions (50.6%) were benign, including fibroadenomas (n=58), benign phyllodes tumor (n=1), cyst (n=5), and benign breast tissues (n=16). The patients’ characteristics and tumor features are presented in **Table 2**.

**Table 2:** The patients’ clinical and demographic characteristics and tumor features.

Characteristics	Benign lesions (n = 80)	Malignant lesions (n = 78)	*P*-value
**Patient characteristics**			
Age (range, y)	35 (17~51)	52 (26~71)	<0.001
Menstrual status			<0.001
Premenopausal	78	42	
Postmenopausal	2	36	
**Lesion characteristics**			
Size (range, mm)	17 (6~80)	24 (11~110)	<0.001
Shape			<0.001
Oval or round	56	24	
Irregular	24	54	
Margin			<0.001
Circumscribed	63	24	
Not circumscribed	17	54	
**DWI parameter**			
ADC (range,×10^-3^mm^2^/s)	1.440 (0.440~2.250)	0.955 (0.680~1.550)	<0.001
**DKI parameter**			
MK (range)	0.530 (0.000~1.907)	1.269 (0.609~2.080)	<0.001
MD (range, ×10^-3^ mm^2^/s)	1.478 (0.723~2.360)	1.049 (0.726~1.508)	<0.001
**tCho peak**	10	41	<0.001
**DCE-MRI parameters**	**Benign lesions (n=46)**	**Malignant lesions (n=17)**	
V_e_(range)	0.671 (0.149~1.000)	0.955 (0.285~0.999)	0.050
K_ep_(range, min^-1^)	0.444 (0.056~1.745)	0.799 (0.330~2.729)	<0.001
K_trans_(range, min^-1^)	0.341 (0.032~1.754)	0.528 (0.132~1.497)	0.019
TIC			
Not enhancement	6	0	
persistent (type I)	30	5	
plateau (type II)	10	11	
washout (type III)	0	1	

### Imaging Parameters for Differentiating Between Malignant and Benign BLs

**Figure 2:**
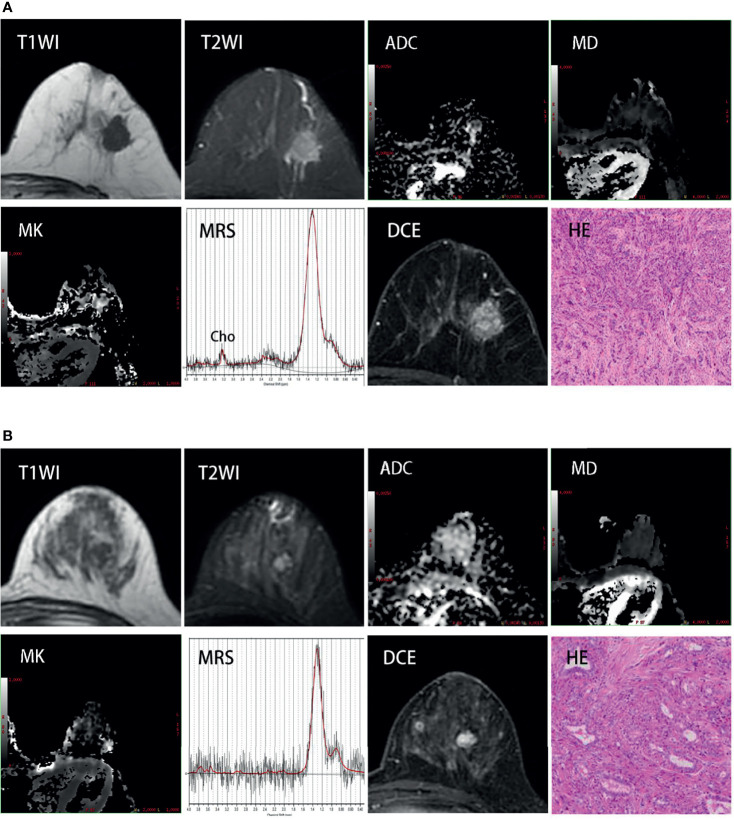
**(A)** Invasive ductal breast carcinoma grade 3 (estrogen receptor-positive, 98%; progesterone receptor-positive, 98%; HER-2-negative, Ki-67-positive, 90%) in a 58 year-old woman. Images show an unregular lesion of decreased T1 signal and increased T2 signal in the left breast. DCE-MRI shows a mass with unregular borders and imhomogenous enhancement. MK map shows increased signal intensity in this region compared with surrounding glandular (mean:1.867); MD map and ADC map both show decreased signal intensity in the same region (mean:0.919×10^-3^mm^2^/sec and 0.635×10^-3^mm^2^/sec); ^1^H-MRS shows a noticeable Cho peak at 3.23 ppm. **(B)** Fibroadenoma in a 35-year-old woman. There is an oval lesion with decreased T1 signal, increased T2 signal, regular borders and homogenous enhancement in the left breast. MK map, MD map and ADC map show non-different signal intensity in this region compared with surrounding glandular(mean: 0.243, 1.93×10^-3^mm^2^/sec and 1.613×10^-3^mm^2^/sec); Cho peak doesn’t appear at 3.23 ppm in ^1^H-MRS.

**Figure 3:**
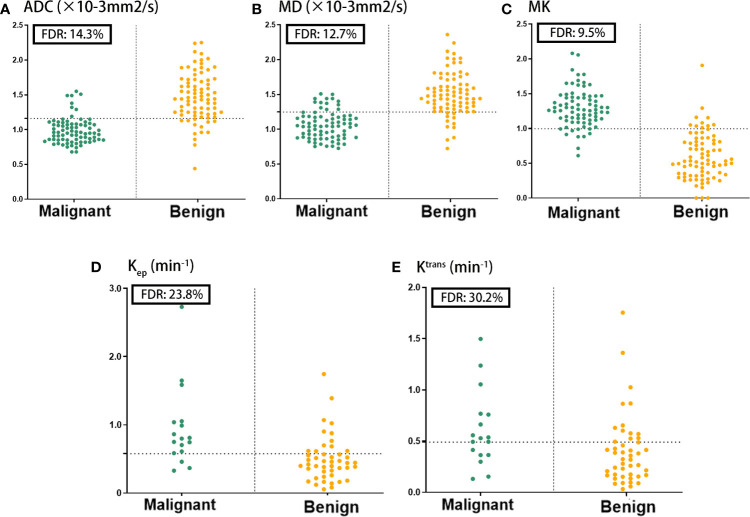
Comparison of the false diagnosis rate (FDR) for ADC **(A)**, MD **(B)**, MK **(C)**, K_ep_ **(D)** and K_trans_ **(E)** parameters in differentiating between benign and malignant breast lesions.

Parametric ADC, DKI, ^1^H-MRS, and DCE-MRI maps were successfully generated for all patients, with two representative cases shown in **Figure 2**. Relative to benign lesions, malignant lesions were associated with lower ADC values ([median 0.955, range 0.680~1.550]×10^-3^ mm^2^/s vs. [median 1.440, range 0.440~2.250]×10^-3^ mm^2^/s; *p*<0.001), lower MD values ([median 1.049, range 0.726~1.508]×10^-3^ mm^2^/s vs. [median 1.478, range 0.723~2.360] ×10^-3^ mm^2^/s; *p*<0.001), higher MK values ([median 1.269, range 0.609~2.080] vs. [median 0.530, range 0.000~1.907]; *p*<0.001), higher K_ep_ value ([median 0.799, range 0.330~2.729] min^-1^ vs. [median 0.444, range 0.056~1.745] min^-1^; *p*<0.001), and higher K^trans^ value ([median 0.528, range 0.132~1.497] min^-1^ vs. [median 0.341, range 0.032~1.754] min^-1^; *p = 0.019*),as shown in **Table 2**. The V_e_ values did not show a significant difference between benign and malignant masses. Based on the AUC values shown in **Table 3**, MK was better for differentiating between malignant and benign lesions (0.952), than ADC (0.902), MD (0.891), tCho (0.766), K_ep_ (0.793) and K^trans^ (0.692). The performance of DKI combined with ADC and DCE-MRI showed the highest AUC compared with other modalities, but there was no significant difference in the diagnostic accuracies of DKI alone or combinations of multiple parameters. Using 0.866 as the MK cut-off value for identifying malignant BLs revealed that samples with values below the cut-off point resulted in a false diagnosis rate of 9.5% for MK, which was lower than the rates for ADC (14.3%), MD (12.7%), tCho (21.9%), K_ep_ (23.8%), and K^trans^ (30.2%), shown in **Figure 3**.

**Table 3:** ROC analysis of the diagnostic performance for MK, MD, ADC, tCho, K_ep_ and K^trans^ alone or in combination for differentiation of malignant and benign lesions.

Multi-parameters	AUC (95% CI)	Cut-off	Sensitivity	Specificity	Accuracy
ADC	0.902 (0.845–0.944)	1.151	89.7% (70/78)	83.7% (67/80)	86.7% (137/158)
MD	0.891 (0.839–0.943)	1.243	83.3% (65/78)	86.2% (69/80)	84.8% (134/158)
MK	0.952 (0.918–0.985)	0.866	97.4% (76/78)	81.2% (65/80)	89.2% (141/158)
tCho	0.766 (0.652–0.857)	–	80.4% (41/51)	72.7% (16/22)	78.1% (57/73)
K_ep_	0.793 (0.668-0.918)	0.568	82.4% (14/17)	73.9% (34/46)	76.2% (48/63)
K^trans^	0.692 (0.548-0.836)	0.487	64.7% (11/17)	71.7% (33/46)	69.8% (44/63)
MK+MD	0.951 (0.866-0.990)	–	88.2% (15/17)	95.7% (44/46)	93.7% (59/63)
MK+MD+ADC	0.957 (0.895-992)	–	94.1% (16/17)	89.1% (41/46)	90.5% (57/63)
ADC+ K_ep_+ K^trans^	0.895 (0.792-0.958)	–	82.4% (14/17)	87.0% (40/46)	85.7% (54/63)
MK+MD + K_ep_+ K^trans^	0.964 (0.884-0.995)	–	100% (17/17)	93.5% (43/46)	95.2% (60/63)
ADC+MK+MD+K_ep_+K^trans^	0.967 (0.888-0.996)	–	100% (17/17)	93.5% (43/46)	95.2% (60/63)

### Imaging Parameters to Predict Prognostic Factors and Molecular Subtype

**Figure 4:**
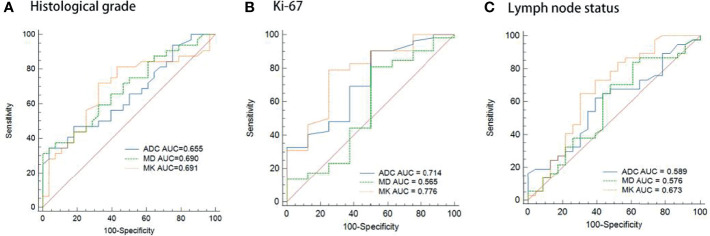
Comparison of the diagnositic performance for ADC, MD and MK in predicting histological grade **(A)**, Ki-67 expression **(B)** and Lymph node status **(C)**.

MK values were significantly higher for high-grade BC than for low-grade BC ([median 1.362, range 0.881~1.843] vs. [median 1.195, range 0.968~1.773]; *p = 0.011*). ADC and MD values were markedly lower for high-grade than for low-grade BC ([median 0.92, range 0.74~1.19]×10^-3^ mm^2^/s vs. [median 0.97,range 0.82~1.55]×10^-3^ mm^2^/s; *p = 0.040;* median 1.034, range 0.757~1.440]×10^-3^ mm^2^/s vs. [median 1.142, range 0.888~1.508]×10^-3^ mm^2^/s; *p = 0.012*) (**Table 4**). MK was superior for predicting the histological grade (AUC: 0.691), over ADC (AUC: 0.655) and MD (AUC: 0.690)(**Figure 4A**). Furthermore, MK was positively correlated with the tumor’s histological grades (r = 0.326, *p* = *0.025*), while both ADC (r = -0.290, *p* = *0.007*) and MD (r = -0.347, *p* = *0.011*) was negatively correlated with the histological grade.

The MK value was significantly higher in BC with high ki-67 expression than those with low Ki-67 expression ([median, 1.288, range, 0.883~1.843]×10^-3^ mm^2^/s vs [median 1.081, range 0.714~1.410]×10^-3^ mm^2^/s; *p* = *0.012*), while both ADC and MD values had a tendency to be lower in high Ki-67 expression BC than in low Ki-67 expression BC (**Table 4**). Moreover, Ki-67 expression was fair correlated with MK (r=0.326, *p = 0.011*), and was better predicted by MK (AUC: 0.776) than by ADC (AUC: 0.714) and MD (AUC: 0.565) (**Figure 4B**).

**Table 4:** Comparison of ADC, MK, MD, V_e_, K_ep_, K^trans^ among different subtypes of breast cancer.

	ADC (n = 60, ×10^-3^ mm^2^/s)	MD (n = 60, ×10^-3^ mm^2^/s)	MK (n = 60)	V_e_ (n = 16)	K_ep_ (n = 16, min^-1^)	K_trans_ (n = 16, min^-1^)
**Histological grade**						
P value	0.040*	0.012*	0.011*	0.999	0.713	0.562
High	0.92,0.74~1.19	1.034,0.757~1.440	1.326,0.881~1.843	0.702,0.549~0.997	0.729,0.368~2.729	0.454,0.154~1.497
Low	0.97,0.82~1.55	1.142,0.888~1.508	1.195,0.968~1.773	0.660,0.285~0.999	0.831,0.330~1.588	0.547,0.132~1.054
**Ki-67**						
P value	0.051	0.557	0.012*	0.439	0.521	0.611
≥14%	0.96,0.74~1.51	1.070,0.757~1.508	1.288,0.883~1.843	0.671,0.468~0.999	0.811,0.330~2.729	0.528,0.154~1.497
<14%	1.09,0.86~1.55	1.126,0.869~1.502	1.081,0.714~1.410	0.549,0.285~0.965	0.751,0.460~0.799	0.414,0.132~0.767
**Lymph node status**						
P value	0.251	0.327	0.025*	0.900	0.704	0.364
Positive	0.96,0.74~1.55	1.052,0.757~1.508	1.300,0.993~1.843	0.671,0.285~0.999	0.799,0.330~2.729	0.528,0.132~1.497
Negative	0.97,0.77~1.19	1.148,0.792~1.502	1.193,0.714~1.777	0.616,0.549~0.924	0.751,0.585~0.811	0.414,0.154~0.537
**ER status**						
P value	0.867	0.224	0.204	0.635	0.492	0.181
Positive	0.96,0.68~1.55	1.104,0.757~1.508	1.257,0.714~1.843	0.743,0.285~0.999	0.772,0.330~1.588	0.429,0.132~1.054
Negative	0.95,0.77~1.29	1.040,0.786~1.440	1.326,0.881~1.660	0.631,0.549~0.924	0.807,0.585~2.729	0.547,0.414~1.497
**PR status**						
P value	0.550	0.679	0.581	0.636	0.492	0.181
Positive	0.94,0.74~1.55	1.087,0.757~1.508	1.262,0.714~1.843	0.743,0.285~0.999	0.772,0.330~1.588	0.429,0.132~1.054
Negative	0.99,0.77~1.49	1.049,0.786~1.440	1.301,0.881~1.660	0.631,0.549~0.924	0.807,0.585~2.729	0.547,0.414~1.497
**HER-2**						
P value	0.637	0.471	0.244	0.262	0.684	0.103
Positive	0.96,0.77~1.1	1.007,0.792~1.440	1.313,1.088~1.777	0.856,0.562~0.999	0.848,0.585~2.729	0.648,0.528~1.497
Negative	0.96,0.74~1.55	1.082,0.757~1.508	1.264,0.714~1.843	0.633,0.285~0.997	0.775,0.330~1.588	0.453,0.132~1.054
**TNBC vs. non-TNBC**						
P value	0.728	0.267	0.558	0.364	0.439	0.704
TNBC	0.97,0.80~1.19	1.043,0.786~1.186	1.336,0.881~1.630	0.613,0.549~0.649	0.863,0.751~1.040	0.557,0.414~0.663
non-TNBC	0.96,0.74~1.55	1.097,0.757~1.508	1.259,0.714~1.843	0.787,0.285~0.999	0.772,0.330~2.729	0.493,0.132~1.497

The bold values and the symbol * are all for marking the Significant statistical difference.

MK was also significantly higher in BC with axillary LN involvement than those without axillary LN involvement ([median, 1.300, range, 0.993~1.843]×10^-3^ mm^2^/s vs [median 1.193, range 0.714~1.777]×10^-3^ mm^2^/s; *p* = *0.025*), while ADC and MD didn’t show significant differences (*p*>0.05) (**Table 4**). In addition, MK presented a fair correlation with lymph node status (*r = 0.291, p = 0.024*), and its predictive ability of lymph node status (AUC: 0.673) was higher than ADC (AUC: 0.589) and MD (AUC: 0.576) (**Figure 4C**).

There were no significant differences regarding MK, MD, or ADC values according to LN involvement, and PR, ER, or TNBC status (**Table 4**). Pharmacokinetic parameters, such as V_e_, K_ep_, and K^trans^, also did not show statistical differences in predicting prognostic factors and molecular subtypes.

### Interobserver Agreement for ADC, MK, MD, V_e_, K_ep_ and K^trans^

The ICC (two-way random model) for the two radiologists’ assessments were 0.963 (95% confidence interval [CI]: 0.950–0.973), 0.978 (95% CI: 0.970–0.984), 0.976 (95% CI: 0.968–0.982), 0.908 (95% CI: 0.852–0.943), 0.950 (95% CI: 0.918–0.969), 0.920 (95% CI: 0.872–0.951) for ADC, MK, MD, Ve, Kep and K^trans^, respectively.

### Construction and Validation of an Optimal XGboost Model for Predicting BC in BiRADS 4 Masses

**Figure 5:**
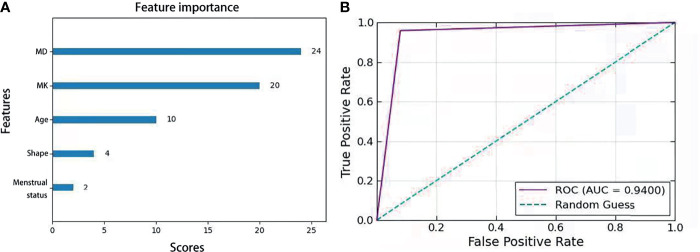
**(A)** Feature importance score in XGboost algorithm model combined with MD, MK, age,shape and menstrual status. **(B)** Receiver operating characteristic curve analysis of the models for BC characterization.

Univariate analysis revealed the following significant risk factors for BC: patient age, menstrual status, lesion size, lesion shape, lesion margin status, ADC, MK, MD, tCho, K_ep_ and K^trans^ values, as shown in **Table 2**. According to lasso regression and single factor ROC value comparison, 5 non-enhanced features including MD, MK, age, shape and menstrual status were selected to be the optimized feature subset to construct a XGboost model, in which MD and MK were the most significant features, with the importance scores of 24 and 20 respectively (**Figure 5A**). This XGboost model exhibited superior diagnostic ability for BC characterization in the training group, with a ROC value of 0.940 (**Figure 5B**). To further verify the predictive reliability of this model for BC diagnosis, 50 BiRADS 4 masses in the validation group were introduced into this model, of which 21 cases were correctly diagnosed as malignant and 22 cases were correctly diagnosed as benign ones, with the diagnostic sensitivity and specificity of 84% and 88%, respectively (**Table 5**).

**Table 5:** Validation of a XGboost model for predicting BC in MRI BiRADS 4.

Biopsy	XGboost model, n (%)	
	Benign	Malignant
**Benign (n=25)**	22 (88%)	3 (12%)
**Malignant (n=25)**	4 (16%)	21 (84%)

## Discussion

This study demonstrated that MK derived from DKI was performed better than MD, ADC, V_e_, K_ep_ and K^trans^ for differentiating between benign and malignant BLs. Also, MK was shown to be more strongly correlated with histological grade, Ki-67 expression and LN status, and was proved to be a promising imaging marker for predicting the clinical and pathological characteristics of BC. Finally, an optimized XGboost model was constructed by combining MD, MK, age, shape and menstrual status, which exhibited superior diagnostic performance for BC characterization and an improved assessment of suspicious breast tumors in BiRADS 4. Overall, we provide a novel and minimally invasive means by using DKI as relevant predictors for diagnosing and determining the microstructural characteristics of BCs.

The rapid proliferation of different cell types makes BC a highly heterogeneous cancer, which may be reflected in the elevated MK values and the concurrently decreased MD and ADC values. MK derived from DKI quantifies the degree that water diffusion deviates from Gaussian diffusion and reflects the tissue complexity, which is considered proportional to the neoplasm’s cellular microstructural heterogeneity and tissue complexity. MD is a corrected diffusion coefficient that removes non-Gaussian bias. In malignant tissues, water molecule diffusion is usually restricted by intracellular, extracellular, and intravascular spaces, as well as by tightened cellular membrane microstructures, leading to lower ADC and MD values. Here, MK was superior for distinguishing between malignant and benign BLs, over MD and ADC. This finding might be explained by ADC relying on an assumption of an ideal Gaussian distribution of unrestricted water diffusion, while the DKI technique assumes that water diffusion follows a non-Gaussian distribution, which is better to explain tissue complexity or physical barriers to diffusion within tissue (cell membranes, organelles, stromal desmoplasia, and so forth) (8). Single-voxel ^1^H-MRS-based tCho peak detection was also evaluated, although it was less effective than MK for differentiating between malignant and benign BLs. The low sensitivity of ^1^H-MRS might be explained by various factors, including the need for high-quality shimming and fat-suppression. Poor shimming results in B_0_ field inhomogeneities that broaden spectral line widths, causing a reduction of SNR and the ability to separate different chemical resonances. Moreover, it also compromises the performance of chemically selective fat and water suppression in localized MRS. Without appropriate fat suppression, lipid sidebands can obscure choline peaks in the spectra. What’s more, breast MRI presents low sensitivity for detecting choline levels in smaller lesions (<10 mm), which limits the applicability of MRS in this model.

This study also revealed higher MK, but lower ADC and MD values, for BC cases that involved high-grade disease and high Ki-67 expression. Similar results have been observed in previous studies (24, 25). Ki-67 expression is a biomarker for cell proliferation, with high expression suggesting increased cellularity, vascular hyperplasia, and necrosis. High-grade tumors are also characterized by active mitosis and the absence of normal glandular architecture, which is correlated positively with high Ki-67 expression (36, 37). These changes reflect BC-related hypercellularity and increased microstructural complexity, leading to higher kurtosis and lower diffusivity. Nevertheless, MK exhibited a strongly correlation with Ki-67 expression and Histological grade, suggesting that DKI is a valuable tool for characterizing BC.

MK was significantly higher in tumors with axillary LN involvement than those without axillary LN involvement in this study, which agrees with the findings by Huang et al. (25) but conflicts with those by Sun et al. (24). This discrepancy might owe to differences in tumor size, as our study involved a greater number of larger lesions, which may tend to be more heterogeneous. This study failed to detect significant differences in any of the imaging parameters according to TNBC or non-TNBC type. This finding might be related to the fact that ER expression inhibits angiogenesis, which might restrict water diffusion in ER-positive BC, while HER2 and PR expression can increase angiogenesis (38). Therefore, ER, PR, and HER2 expression might influence angiogenesis by regulating vascular endothelial growth factor production at different levels in BC. Nevertheless, tumor heterogeneity also likely contributes to the lack of a clear relationship between the BC subtype and imaging parameters.

DCE-MRI, making use of Tofts two-compartment model, quantifies the contrast agent exchange between the intravascular and the interstitial space, providing measurements of tumor blood flow, the microvasculature, and capillary permeability. The pharmacokinetic parameters can potentially improve the differentiation of benign and malignant breast tumors and distinguish different breast cancer subtypes (39). This study revealed that there were significant differences in K^trans^ and K_ep_ between benign and malignant tumors, which agree with the previous study by Li et al. (6). However, no significant differences were observed between pharmacokinetic parameters and prognostic factors of BCs, and the diagnostic efficiency of pharmacokinetic parameters was also lower than that of MD and MK. The possible reason might be the fact that there were only two BCs which exhibited a wash-out type of dynamic enhancement pattern.

XGboost algorithm has been widely used in medical fields, such as Chronic Kidney Disease Diagnosis (30), COVID-19 (40), etc. Hou et al. (31) compared the diagnostic efficiency of the logistic regression model, SAPS-II score prediction model and XGboost algorithm model in predicting 30-days mortality for MIMIC-III patients, and found that the XGboost model performed the best, indicating its great potential in medical applications. As can be seen in **Figure 5** and **Table 5**, the optimized XGboost model in our study exhibited superior diagnostic ability for BC characterization in both the test group and the validation group. In particular, this model improved the diagnostic specificity of BiRADS 4 tumors, suggesting its potential usefulness in reducing the number of unnecessary biopsies, as well as reducing anxiety of patients and waste of medical resources in the long term.

Our study had some limitations. First, the sample size was relatively small, and only a few patients had PR-positive tumors or the TNBC type. Thus, using a larger sample with multiple histological BC types may yield a more accurate estimate. Second, the low spatial resolution of DWI might lead to inaccurate measurements of small benign lesions (< 1 cm). Hence, an improved high-resolution sequence for DWI might be required to detect small lesions. Third, all parameters were calculated on the same MR scanner, and our findings might be specific to the sequences we used.

In conclusion, our study demonstrated that DKI is promising for breast cancer diagnosis and prognostic factor assessment. An optimized XGboost model that included DKI, age, shape and menstrual status is effective to improve the diagnostic specificity of BiRADS 4 masses, thereby preventing unnecessary biopsies and optimizing personalized diagnosis and treatment. However, a multicenter prospective study with a larger cohort should be performed in the near future to validate these results.

## Data Availability Statement

The original contributions presented in the study are included in the article/supplementary material. Further inquiries can be directed to the corresponding author.

## Ethics Statement

Written informed consent was obtained from the individual(s), and minor(s)’ legal guardian/next of kin, for the publication of any potentially identifiable images or data included in this article.

## Author Contributions

WT and HZ designed the experiments. TQ, WT, and HZ analyzed the data. WT, HZ, XC, and HNZ contributed to MRI data acquisition. WT and HZ wrote the paper. YL and RW supervised the experiment. All authors contributed to the article and approved the submitted version.

## Funding

This study was supported by grants from the National Natural Science Foundation of China (82071973, 82020108016, 81471729) and Natural Science Foundation of Guangdong Province (2020A1515011022).

## Conflict of Interest

The authors declare that the research was conducted in the absence of any commercial or financial relationships that could be construed as a potential conflict of interest.

## Publisher’s Note

All claims expressed in this article are solely those of the authors and do not necessarily represent those of their affiliated organizations, or those of the publisher, the editors and the reviewers. Any product that may be evaluated in this article, or claim that may be made by its manufacturer, is not guaranteed or endorsed by the publisher.

## Abbreviations

BC, breast cancer; tCho, total choline; BLs, breast lesions; LN, lymph node; ER, estrogen receptor; PR, progesterone receptor; HER2, human epidermal growth factor receptor 2; lasso, the selection operator algorithm; FSE-XL, fast spin echo eXel; FRFSE-XL, fast relaxation fast spin echo eXcel; EPI, Echo Planar Imaging; VIBRANT, volume imaging for breast assessment; PRESS, point-resolved spectroscopy; SNR, signal-noise radio; STIR, Short inversion-Time Inversion Recovery.

## References

[1] Sung H Ferlay J Siegel RL Laversanne M Soerjomataram I Jemal A. Global Cancer Statistics 2020: GLOBOCAN Estimates of Incidence and Mortality Worldwide for 36 Cancers in 185 Countries. CA: Cancer J Clin (2021) 71(3):209–49. doi: 10.3322/caac.21660 33538338

[2] Holm J Eriksson L Ploner A Eriksson M Rantalainen M Li J. Assessment of Breast Cancer Risk Factors Reveals Subtype Heterogeneity. Cancer Res (2017) 77(13):3708–17. doi: 10.1158/0008-5472.Can-16-2574 28512241

[3] Perou CM Sørlie T Eisen MB van de Rijn M Jeffrey SS Rees CA. Molecular Portraits of Human Breast Tumours. Nature (2000) 406(6797):747–52. doi: 10.1038/35021093 10963602

[4] Wei F Chen W Lin X. Efficacy and Safety of Adjuvant Endocrine Therapy in Premenopausal Patients With Early-Stage Hormone Receptor-Positive Breast Cancer: A Meta-Analysis of Randomized Controlled Trials. Breast J (2019) 25(6):1297–9. doi: 10.1111/tbj.13465 31301082

[5] Kuhl CK. The Changing World of Breast Cancer: A Radiologist's Perspective. Invest Radiol (2015) 50(9):615–28. doi: 10.1097/RLI.0000000000000166 PMC4623842 26083829

[6] Li L Wang K Sun X Wang K Sun Y Zhang G. Parameters of Dynamic Contrast-Enhanced MRI as Imaging Markers for Angiogenesis and Proliferation in Human Breast Cancer. Med Sci Monit (2015) 21:376–82. doi: 10.12659/MSM.892534 PMC4324575 25640082

[7] Crich SG Terreno E Aime S. Nano-Sized and Other Improved Reporters for Magnetic Resonance Imaging of Angiogenesis. Adv Drug Deliv Rev (2017) 119:61–72. doi: 10.1016/j.addr.2017.08.004 28802567

[8] Rahbar H Partridge SC. Multiparametric MR Imaging of Breast Cancer. Magn Reson Imaging Clin N Am (2016) 24(1):223–38. doi: 10.1016/j.mric.2015.08.012 PMC4672390 26613883

[9] Lee J Kim SH Kang BJ. Pretreatment Prediction of Pathologic Complete Response to Neoadjuvant Chemotherapy in Breast Cancer: Perfusion Metrics of Dynamic Contrast Enhanced MRI. Sci Rep (2018) 8(1):9490. doi: 10.1038/s41598-018-27764-9 29934524 PMC6014994

[10] Liu F Wang M Li H. Role of Perfusion Parameters on DCE-MRI and ADC Values on DWMRI for Invasive Ductal Carcinoma at 3.0 Tesla. World J Surg Oncol (2018) 16(1):239. doi: 10.1186/s12957-018-1538-8 30577820 PMC6303963

[11] Pinker K Bogner W Baltzer P Gruber S Bickel H Brueck B. Improved Diagnostic Accuracy With Multiparametric Magnetic Resonance Imaging of the Breast Using Dynamic Contrast-Enhanced Magnetic Resonance Imaging, Diffusion-Weighted Imaging, and 3-Dimensional Proton Magnetic Resonance Spectroscopic Imaging. Invest Radiol (2014) 49(6):421–30. doi: 10.1097/rli.0000000000000029 24566292

[12] Prvulovic Bunovic N Sveljo O Kozic D Boban J. Is Elevated Choline on Magnetic Resonance Spectroscopy a Reliable Marker of Breast Lesion Malignancy? Front Oncol (2021) 11:610354. doi: 10.3389/fonc.2021.610354 34567998 PMC8462297

[13] Roknsharifi S Fishman MDC Agarwal MD Brook A Kharbanda V Dialani V. The Role of Diffusion Weighted Imaging as Supplement to Dynamic Contrast Enhanced Breast MRI: Can it Help Predict Malignancy, Histologic Grade and Recurrence? Acad Radiol (2019) 26(7):923–9. doi: 10.1016/j.acra.2018.09.003 30293819

[14] Sharma U Agarwal K Hari S Mathur SR Seenu V Parshad R. Role of Diffusion Weighted Imaging and Magnetic Resonance Spectroscopy in Breast Cancer Patients With Indeterminate Dynamic Contrast Enhanced Magnetic Resonance Imaging Findings. Magn Reson Imaging (2019) 61:66–72. doi: 10.1016/j.mri.2019.05.032 31128225

[15] Suo S Cheng F Cao M Kang J Wang M Hua J. Multiparametric Diffusion-Weighted Imaging in Breast Lesions: Association With Pathologic Diagnosis and Prognostic Factors. J Magn Reson Imaging (2017) 46(3):740–50. doi: 10.1002/jmri.25612 28139036

[16] Yuan C Jin F Guo X Zhao S Li W Guo H. Correlation Analysis of Breast Cancer DWI Combined With DCE-MRI Imaging Features With Molecular Subtypes and Prognostic Factors. J Med Syst (2019) 43(4):83. doi: 10.1007/s10916-019-1197-5 30810823

[17] Marko NF Weil RJ. Non-Gaussian Distributions Affect Identification of Expression Patterns, Functional Annotation, and Prospective Classification in Human Cancer Genomes. PloS One (2012) 7(10):e46935. doi: 10.1371/journal.pone.0046935 23118863 PMC3485292

[18] Jensen JH Helpern JA Ramani A Lu H Kaczynski K. Diffusional Kurtosis Imaging: The Quantification of non-Gaussian Water Diffusion by Means of Magnetic Resonance Imaging. Magn Reson Med (2005) 53(6):1432–40. doi: 10.1002/mrm.20508 15906300

[19] Christou A Ghiatas A Priovolos D Veliou K Bougias H. Accuracy of Diffusion Kurtosis Imaging in Characterization of Breast Lesions. Br J Radiol (2017) 90(1073):20160873. doi: 10.1259/bjr.20160873 28383279 PMC5605109

[20] Wu D Li G Zhang J Chang S Hu J Dai Y. Characterization of Breast Tumors Using Diffusion Kurtosis Imaging (DKI). PloS One (2014) 9(11):e113240. doi: 10.1371/journal.pone.0113240 25406010 PMC4236178

[21] Li C Chen M Wan B Yu J Liu M Zhang W. A Comparative Study of Gaussian and non-Gaussian Diffusion Models for Differential Diagnosis of Prostate Cancer With in-Bore Transrectal MR-Guided Biopsy as a Pathological Reference. Acta radiologica (Stockholm Sweden: 1987) (2018) 59(11):1395–402. doi: 10.1177/0284185118760961 29486596

[22] Zhang J Chen X Chen D Wang Z Li S Zhu W. Grading and Proliferation Assessment of Diffuse Astrocytic Tumors With Monoexponential, Biexponential, and Stretched-Exponential Diffusion-Weighted Imaging and Diffusion Kurtosis Imaging. Eur J Radiol (2018) 109:188–95. doi: 10.1016/j.ejrad.2018.11.003 30527302

[23] Yuan ZG Wang ZY Xia MY Li FZ Li Y Shen Z. Comparison of Diffusion Kurtosis Imaging Versus Diffusion Weighted Imaging in Predicting the Recurrence of Early Stage Single Nodules of Hepatocellular Carcinoma Treated by Radiofrequency Ablation. Cancer Imaging: Off Publ Int Cancer Imaging Soc (2019) 19(1):30. doi: 10.1186/s40644-019-0213-9 PMC6542145 31142356

[24] Sun K Chen X Chai W Fei X Fu C Yan X. Breast Cancer: Diffusion Kurtosis MR Imaging-Diagnostic Accuracy and Correlation With Clinical-Pathologic Factors. Radiology (2015) 277(1):46–55. doi: 10.1148/radiol.15141625 25938679

[25] Huang Y Lin Y Hu W Ma C Lin W Wang Z. Diffusion Kurtosis at 3.0T as an *In Vivo* Imaging Marker for Breast Cancer Characterization: Correlation With Prognostic Factors. J Magn Reson Imaging (2019) 49(3):845–56. doi: 10.1002/jmri.26249 30260589

[26] Liberman L Menell JH. Breast Imaging Reporting and Data System (BI-RADS). Radiol Clin North Am (2002) 40(3):409–30. doi: 10.1016/s0033-8389(01)00017-3 12117184

[27] Magny SJ Shikhman R Keppke AL. Breast Imaging Reporting and Data System. Treasure Island (FL: StatPearls (2021). 29083600

[28] Leithner D Wengert G Helbich T Morris E Pinker K. MRI in the Assessment of BI-RADS(R) 4 Lesions. Top Magn Reson Imaging (2017) 26(5):191–9. doi: 10.1097/RMR.0000000000000138 28961568

[29] ACM Press the 22nd ACM SIGKDD International Conference - San Francisco, California, USA (2016.08.13-2016.08.17). Proceedings of the 22nd ACM SIGKDD International Conference on Knowledge Discovery and Data Mining - KDD '16 - XGBoost. p. 785–94. doi: 10.1145/2939672.2939785

[30] Ogunleye A Wang QG. XGBoost Model for Chronic Kidney Disease Diagnosis. IEEE/ACM Trans Comput Biol Bioinform (2020) 17(6):2131–40. doi: 10.1109/TCBB.2019.2911071 30998478

[31] Hou N Li M He L Xie B Wang L Zhang R. Predicting 30-Days Mortality for MIMIC-III Patients With Sepsis-3: A Machine Learning Approach Using XGboost. J Transl Med (2020) 18(1):462. doi: 10.1186/s12967-020-02620-5 33287854 PMC7720497

[32] Sood N Nigam JS Yadav P Rewri S Sharma A Omhare A. Comparative Study of Cytomorphological Robinson’s Grading for Breast Carcinoma With Modified Bloom-Richardson Histopathological Grading. Pathol Res Int (2013) 2013:146542. doi: 10.1155/2013/146542 PMC3800647 24187646

[33] Hammond ME Hayes DF Wolff AC Mangu PB Temin S. American Society of Clinical Oncology/College of American Pathologists Guideline Recommendations for Immunohistochemical Testing of Estrogen and Progesterone Receptors in Breast Cancer. J Oncol Pract (2010) 6(4):195–7. doi: 10.1200/jop.777003 PMC2900870 21037871

[34] Wolff AC Hammond MEH Allison KH Harvey BE Mangu PB Bartlett JMS. Human Epidermal Growth Factor Receptor 2 Testing in Breast Cancer: American Society of Clinical Oncology/College of American Pathologists Clinical Practice Guideline Focused Update. J Clin Oncol: Off J Am Soc Clin Oncol (2018) 36(20):2105–22. doi: 10.1200/jco.2018.77.8738 29846122

[35] Focke CM Bürger H van Diest PJ Finsterbusch K Gläser D Korsching E. Interlaboratory Variability of Ki67 Staining in Breast Cancer. Eur J Cancer (Oxford England: 1990) (2017) 84:219–27. doi: 10.1016/j.ejca.2017.07.041 28829990

[36] Healey MA Hirko KA Beck AH Collins LC Schnitt SJ Eliassen AH. Assessment of Ki67 Expression for Breast Cancer Subtype Classification and Prognosis in the Nurses’ Health Study. Breast Cancer Res Treat (2017) 166(2):613–22. doi: 10.1007/s10549-017-4421-3 PMC6995281 28791482

[37] Kamranzadeh H Ardekani RM Kasaeian A Sadighi S Maghsudi S Jahanzad I. Association Between Ki-67 Expression and Clinicopathological Features in Prognosis of Breast Cancer: A Retrospective Cohort Study. J Res Med Sci: Off J Isfahan Univ Med Sci (2019) 24:30. doi: 10.4103/jrms.JRMS_553_18 PMC6521610 31143231

[38] Suo S Zhang K Cao M Suo X Hua J Geng X. Characterization of Breast Masses as Benign or Malignant at 3.0T MRI With Whole-Lesion Histogram Analysis of the Apparent Diffusion Coefficient. J Magn Reson Imaging (2016) 43(4):894–902. doi: 10.1002/jmri.25043 26343918

[39] Pinker K Helbich TH Morris EA. The Potential of Multiparametric MRI of the Breast. Br J Radiol (2017) 90(1069):20160715. doi: 10.1259/bjr.20160715 27805423 PMC5605035

[40] McDonald SA Medford RJ Basit MA Diercks DB Courtney DM. Derivation With Internal Validation of a Multivariable Predictive Model to Predict COVID-19 Test Results in Emergency Department Patients. Acad Emerg Med (2021) 28(2):206–14. doi: 10.1111/acem.14182 PMC7753649 33249683

